# Posterior Reversible Encephalopathy Syndrome Induced by an Acute Postinfectious Glomerulonephritis

**DOI:** 10.1155/2021/8850092

**Published:** 2021-03-03

**Authors:** Jamal Ouachaou, Ilyass Laaribi, Mohammed Maarad, Ikram Zaid, Rajae Alkouh, Houssam Bkiyar, Brahim Housni

**Affiliations:** Intensive Care Unit, Mohammed VI University Hospital Center, Faculty of Medicine and Pharmacy of Oujda, Mohammed I University, Oujda, Morocco

## Abstract

Posterior reversible encephalopathy (PRES) is a rare but a serious disease that affects the central nervous system. PRES is responsible for various but nonspecific neurological symptoms, including confusion, coma, and seizures as well as visual disturbances. Diagnosis is made using cerebral MRI which typically shows at the early stage, bilateral symmetrical parietooccipital hyperintensities on T2 and fluid-attenuated inversion recovery (FLAIR) sequences. *Case study*. In this article, we base our research on a case study that includes, as a population sample, a 9-year-old boy who suffers from an acute postinfectious glomerulonephritis and arterial hypertension. Two days before diagnosis, he developed confusion with generalized tonic-clonic attacks. His blood pressure was 180/80 mmHg. A cerebral computed tomography made in emergency showed cerebral edema. It was supplemented by magnetic resonance imaging which revealed cortical and posterior cortical lesions which appear as hypointense on T1 and hyperintense on T2 and Flair. An MRI control was performed 40 days later which shows a clear improvement of the occipital lesions. PRES is a radioclinical syndrome characterized by the association of variable neurological signs which reversibility is conditioned by the early diagnosis and the correction of the contributing factors.

## 1. Introduction

Reversible posterior encephalopathy syndrome (RPES) is a clinocoradiological diagnosis characterized by the combination of neurological clinical signs and posterior bilateral cerebral edema, in neuroimaging, which is usually reversible [1]. It is popularly diagnosed for adults and children [2].

The main symptoms are the combination of headache, vomiting, alertness disorders, seizures, visual problems, and focal neurological signs.

Neuroimaging shows multifocal lesions that are predominant in the posterior area of the cerebral hemispheres mainly in the parietooccipital one.

Among the common causes behind this disease, we can refer to hypertensive encephalopathy, renal insufficiency, immunosuppressive medication, infections, and systemic diseases.

In our study, we report a case study of a child hospitalized for consciousness disorders followed by hypertensive peaks as part of acute postinfectious glomerulonephritis.

## 2. Observation

The 9-year-old child under study has suffered from nontreated angina 15 days ago. He was hospitalized in the pediatric emergency room. The symptoms revealed by this case study are the following: afebrile consciousness disorders developed in two days and convulsive attacks without vomiting. The clinical exam found a sleepy agitated child without sensory-motor deficit. Moreover, the blood pressure reached 180/80 mmHg, and his diuresis was maintained to 1.2 ml/kg/h. The urine test strip showed some traces of blood. The rest of the clinical exam found minor facial edema.

For biology: creatinine at 28 mmol/L, urea at 2 mmol/L, and proteinuria at 34 mg/24 h, for this atypical case of postinfectious acute glomerulonephritis and after elimination of hemolytic and uremic syndrome, the C3 and ASLO measurements were done. The values were successively 0.45 g/L and 650 U/ml for normal levels between 0.65-1.47 g/L and <500 U/ml. The treatment was symptomatic with antiepileptic medication and intravenous antihypertensive drugs (sodium valproate and nicardipine with syringe driver) as well as the oral treatment such as (nicardipine 25 mg) until blood pressure was readjusted, in addition to antibiotic therapy (amoxicillin + clavulanic acid). The outcome of this therapy was favorable after 48 h of treatment, and the patient regained consciousness and was permitted to quit the hospital after 6 days of hospitalization.

**Figure 1:**
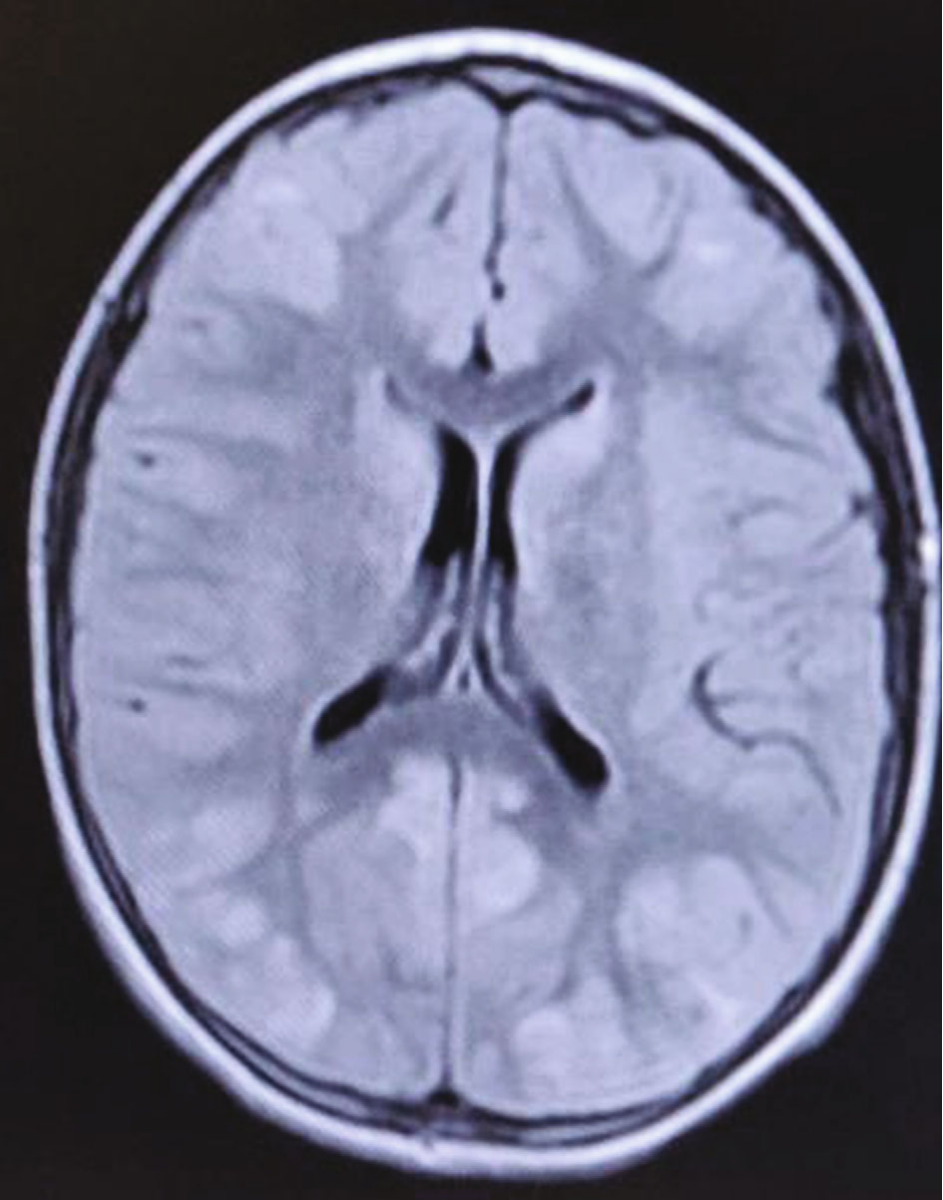
A cerebral magnetic resonance imaging: the main section shows cortical and posterior cortical lesions in hyper signal in T2.

Moreover, an urgent cerebral tomogram is conducted and which has shown cerebral edema. In addition to presenting the clinical diagnosis of the case study, we have also added a magnetic resonance imaging which demonstrates both cortical and posterior subcortical lesions in the form of a hyposignal in T1 and a hypersignal in T2 and Flaire (Figure 1). An MRI checking was done after 40 days and which reveals a significant occipital lesions' improvement.

## 3. Discussion

The PRES was initiated the first time in 1996 by Hinchey et al. [3]. It was named reversible posterior leukoencephalopathy based on a set of 15 subjects which had presented the same clinical and neuroradiological characteristics.

The primary clinical picture is variable, ranging from simple headaches with vomiting to status epilepticus that requires intensive care. For our study, the symptomatology started with confusion and seizure.

In other situations, renal failure can be implicated in the development of PRES particularly when it is due to a glomerulopathy during systemic diseases requiring the use of immunosuppressive therapy.

In most cases, the concerned patients present an unbalanced HBP (high blood pressure) which makes PRES considered as a particular type of hypertensive encephalopathy.

It is crucial to note that PRES can accure without high blood pressure disorders. There are several factors that can be associated with the progression of PRES. Also, in our observation, the child had hypertension and glomerular nephropathy.

The PRES pathophysiology remains hardly understood. Hence, the main hypothesis is based on the existence of an exceeding vascular tone's self-regulating capacities at the cerebral level, in the context of severe hypertension, which is linked to an imbalance between vasoconstrictor and vasodilator substances at the origin of cerebral vasodilatation with cerebral hypoperfusion, endothelial dysfunction, blood-brain barrier breakdown, and capillary leak syndrome leading to cerebral edema [4].

The sympathetic innervation, which is protective in this context, is less in the vertebrobasilar circulation which can explain the preferential posterior localization of lesions [5]. Some direct toxic symptoms of immunosuppressive treatments have also impacted the vascular endothelium [3–6]. For others, it is due to vasoconstriction mechanisms with ischemic phenomena, prompted by both the immune and endothelial activation state responsible for vasogenic cerebral edema [7]. the brain CT scan can be normal or demonstrates bilateral hypodense lesions. Also, it can show parietooccipital subcortical lesions [8].

Among the most advantageous diagnostic tools, MRI is the most suitable exam that helps following disease progression. For instance, lesions are often subcortical, bilateral, and symmetrical in the parietooccipital areas. However, cortical lesions are also possible but rare. Brain stem and cerebellum damages are widespread. These lesions are hypointense T1 with a discreet cortical enhancement after gadolinium injection, demonstrating the blood-brain barrier breakdown. The T2-weighted sequences and especially the Flair sequence appears are the most efficient ones and show hyperintensities areas.

Typically, MRI shows hyperintensity on diffusion sequences with increased apparent diffusion coefficient (ACD), indicating vasogenic edema. On the other hand, cytotoxic edema, related to ischemia, is hyperintense with decreased diffusion coefficient [8]. Magnetic resonance angiography enables the elimination of cerebral venous sinus thrombosis (CVST) which is the main differential diagnosis. When done early, it can show a vasospasm, very evocative of the diagnosis [8].

From a radiological perspective, MRI mainly shows, during PRES, bilateral subcortical hyperintensive areas of the white matter, with parietal and occipital lobe predominance. However, they also affect the cerebellum and brain stem on the T2 and Flair sequences. Nevertheless, the cortex may also be involved, and therefore, the initial title reversible posterior leukoencephalopathy has gradually been replaced by the term—reversible posterior encephalopathy [9]. The diffusion and CDA sequences could have a prognostic interest by differentiating vasogenic edema, usually found during PRES, from cytotoxic edema which indicates potentially irreversible lesions [10, 11].

The atypical MRI finding for PRES is observed as low- to equi-intensity signals under T1 and high-intensity signals in enhanced T2-weighted MRI images, which become more apparent on FLAIR images, and low- to equi-intensity signals are observed on diffusion-weighted images (DWI), and high-intensity signals are seen on the apparent diffusion coefficient (ADC) maps, and these findings are thought to be principally due to vasogenic edema.

In a nutshell, MRI is a noninvasive and reliable method for both the diagnosis and the follow-up of cerebral edema and vasospasm during PRES. Other brain imaging techniques can be used. However, magnetic resonance spectroscopy (MRS) would have outstanding prognostic value in the acute phase of the PRES [12]. This test can identify early transient disorders of aerobic energy metabolism, such as a high level of lactate production. It may also exhibit an increase in choline and creatine levels or a minimal decrease in *N*-acetyl- aspartate levels, which indicates brain tissue damage in PRES [13]. Cerebral scintigraphy or single-photon emission computed tomography (SPECT) most often displays images of hyperperfusion in the acute phase and hypoperfusion in the late phase [14].

As it is shown in our observation, PRES evolution is usually favorable under the adapted treatment which leads to both the resolution of neurological signs and the regression of neuroradiological abnormalities, usually in less than 15 days.

Treatment is essentially based on antihypertensive and anticonvulsant treatments, stopping the offending drugs and management of systemic disease relapse.

## 4. Conclusion and “Take-Away”

PRES syndrome should be suspected in front of a child with a (sub)acute disorder, including headache and convulsions, with evocative posterior radiological images, without forgetting to search for hypertension and the contributory factors.

### 4.1. Patient's Perspective

The parents expressed their satisfaction with hospital care, and they provided the department with appreciation words.

The MRI control test confirmed the clinical and radiological evolution of the patient.

## Data Availability

The data used to support the findings of the study can be available upon request.
